# China Promotes Sanming’s Model: A National Template for Integrated Medicare Payment Methods

**DOI:** 10.5334/ijic.7011

**Published:** 2023-05-11

**Authors:** Zhengdong Zhong, Qiang Yao, Shanquan Chen, Junnan Jiang, Kunhe Lin, Yifan Yao, Li Xiang

**Affiliations:** 1 School of Medicine and Health Management, Huazhong University of Science and Technology, Wuhan, China; 2 School of Political Science and Public Administration, Wuhan University, Wuhan, China; 3 Department of Psychiatry, University of Cambridge, Cambridge CB2 3EB, United Kingdom; 4 School of Public Administration, Zhongnan University of Economics and Law, Wuhan, China

**Keywords:** Sanming model, Integrated Medicare Payment Methods, Global budget, Medical insurance fund balance, Distribution of doctors’ salary

## Abstract

**Introduction:**

China is promoting integrated care. However, incomplete payment methods led to medical insurance overspending and intensified service fragmentation. Sanming implemented Integrated Medicare Payment Methods (IMPM) in October 2017, which integrates multi-level payment policies. Sanming’s IMPM works well and has been promoted by the Chinese government. Therefore, in this paper, we aim to systematically analyze Sanming’s IMPM, and conduct preliminary evaluations of Sanming’s IMPM.

**Policy Description:**

IMPM integrates two levels of policy that are implemented simultaneously: (1) The payment policy for healthcare providers refers to how to calculate the global budget (GB) of the medical insurance fund paid to the healthcare providers and the policy guidance for the healthcare providers on how to use GB. (2) The payment policy for medical personnel refers to the adjustment of the evaluation index of the annual salary system (ASS) according to the IMPM’s purpose and the payment policy that adjust pay levels based on performance.

**Discussion and lessons learned:**

After the IMPM reform, county hospitals (CHs) may reduce over-providing dispensable healthcare, and cooperation between hospitals may increase. The policy guidance (Determining GB according to population; Medical insurance balance can be used for doctors’ salary, cooperation between hospitals, and promotion of residents’ health; Adjusting ASS assessment indicators according to IMPM purposes) increases CHs’ motivation to promote balances of medical insurance fund by cooperating with primary healthcare and increasing health promotion actions.

**Conclusion:**

As a model promoted by the Chinese government, the specific policies of Sanming’s IMPM are better matched with policy goals, which may be more conducive to promoting medical and health service providers to pay more attention to cooperation among medical institutions and population health.

## Introduction (comprising background and problem statement)

Countries around the world have proposed the direction of reform to establish integrated care [1,2]. International experience shows that the integrated health service delivery system is an effective means to improve the performance of the healthcare service system [1], and medical insurance payment plays a key role in the integrated health service system [3,4,5]. However, until 2016, China’s medical insurance payment has been dominated by Fee-For-Service (FFS), which has resulted in a fragmented, and treatment-focused healthcare delivery system to a certain extent [6,7,8]. There is a lack of cooperation, and independent funding between hospitals at different levels [6]. To maximize benefits, large hospitals continue to expand resources [6]. Chinese patients are more and more inclined to go to large hospitals for treatment, which causes serious waste of resources and leads to rapid growth in medical costs [9].

International studies have shown that the integration of health service systems is a combination of multiple factors [10,11,12]. Ways to promote integrated care include integrating the health care service system and reforming the health payment system, etc. [13] At the practice level in China, China’s National Health Commission began to vigorously promote medical alliances and carried out the reform of integrated payment for medical alliances in 2016 to reduce the waste of resources [14,15]. The Integrated County Healthcare Coalition (ICHC) is one of four medical alliances in China [15]. ICHC reform integrates county-level hospitals (CHs), township hospitals (THs), and village health clinics (HCs) in rural areas [16,17]. ICHC reform can reduce disorderly competition among hospitals and reduce integration costs. The organizational structure of ICHC in different regions of China is nearly the same. However, the integrated payment for ICHC in various regions of China is not perfect, which leads to medical insurance overruns and insufficient institutional cooperation [15,16]. However, at the research level in China, current research mainly focuses on how ICHC promotes integration but does not focus on the key role of integrated payments [14,15,16].

In this context, China’s Sanming implemented the Integrated Medicare Payment Methods (IMPM) reform on ICHC [18]. Sanming’s IMPM aims to save medical insurance funds, promote cooperation among medical institutions and promote residents’ health. Sanming’s IMPM was first implemented in Youxi County in October 2017, and the IMPM is still being implemented by 2023 (Only the assessment variables and level have slightly changed). IMPM integrates two levels of policy that are implemented simultaneously: (1) The payment policy for healthcare providers (i.e. ICHC) refers to how to calculate global budget (GB) of the medical insurance fund paid to the ICHC and the policy guidance for the ICHC on how to use GB. (2) The payment policy for medical personnel refers to the adjustment of the evaluation index of the annual salary system (ASS) according to the IMPM’s purpose and the payment policy that adjust pay levels based on performance. The reform in Sanming has attracted the attention of China’s top leaders, and the Chinese government issued a document to promote the Sanming model throughout China in November 2021 [19].

Therefore, this paper aims to systematically analyze Sanming’s IMPM and provide preliminary evidence of the effect of the IMPM. We hope to provide a reference for other low- and middle-income countries similar to China with limited funds to implement integrated payments.

## Description of the IMPM in Sanming

### The payment policies for ICHC in IMPM

IMPM’s payment policy for healthcare providers consists of two main parts: how to calculate GB of the medical insurance fund paid to the ICHC and the policy guidance for the ICHC on how to use GB.

First, the GB of IMPM is different from the previous GB for a single institution, the GB of IMPM is the sum of the total medical insurance of all hospitals in ICHC, and the GB of IMPM is based on the county’s total population multiplied by the annual fixed insurance premium. The fixed insurance premium is calculated based on the health needs of the population, the level of medical technology development, and the price level, so the GB of IMPM usually increases. At the beginning of each year, after the Healthcare Security Administration draws the medical insurance fund for the private hospital and the risk adjustment fund, all the remaining total medical insurance funds are allocated to ICHC.

Second, the policy guidance on how to use GB consists of four main types of guidance:

1. **Owning the over-spending**-If the actual medical insurance expenditure of ICHC at the end of the year is higher than the total medical insurance fund at the beginning of the year (that is, the medical insurance fund over-spending), the part exceeding the total medical insurance fund shall be borne by ICHC and the Healthcare Security Administration will not pay any additional medical insurance funds to ICHC. **Remaining the balance-**If the actual medical insurance expenditure of ICHC at the end of the year is less than the total medical insurance fund at the beginning of the year and the ICHC has passed the assessment (that is, the medical insurance fund balance), the balance shall all belong to the medical service income of ICHC.
2. ICHC can withdraw a certain percentage of the balance (16% in 2018) as the performance of all ICHC medical staff’s salary incentives. This means that the healthier the regional population, the lower the actual cost of medical services, the more the ICHC benefits, and the higher the ICHC’s medical staff wages.
3. ICHC may use the balance for actions that facilitate cooperation among CHs, THs, and HCs, which aims to promote cooperation among different hospitals.
4. ICHC may use the balance to carry out health promotion programs other than basic public health services, which aim to promote health and prevent disease.

### The payment policies for medical personnel in IMPM

As of 2015, Sanming has implemented ASS for medical staff (including public hospital directors). The payment policy for medical personnel in IMPM refers to:

1. To cooperate with the payment policy for medical service providers of IMPM, Sanming has added indicators related to IMPM’s reform purpose in ASS. For example, Sanming has added indicators for directors’ ASS, such as medical insurance fund balance, health promotion, cooperation with medical institutions (the proportion of the number of healthcare outpatient visits in primary healthcare institutions), etc. Sanming has added indicators for doctors such as health education, health management, and promotion of institutional cooperation (for example, CHs doctors go to THs for consultations).
2. Sanming established the online intelligent supervision system matching the IMPM target to control medical personnel’s behavior and avoid negative effects. Sanming supervises assessment indicators of IMPM through the online intelligent supervision system, and the results of performance assessment will directly affect the salary level of medical personnel. If hospitals and doctors perform well, the scores will be raised accordingly, and the annual salaries of the medical staff of public hospitals will be raised accordingly.

## Comparison of Sanming’s IMPM with other areas of China

In China, the medical insurance administration pays the funds to the hospitals first, and then the hospitals allocate the funds to the doctors, unlike in some other countries where the medical insurance administration pays the funds directly to the doctors. Meanwhile, ICHC reform in Sanming is not much different from that in other areas of China. Therefore, this study mainly analyzes the differences between Sanming IMPM and other regions in China.

The goal of integrated payment policies in Sanming and other parts of China is to save medical insurance funds, promote cooperation among medical institutions and promote residents’ health. In practical policy, however, there are big differences between Sanming and the rest of China (See Table 1 for details).

**Table 1:** Comparison of Sanming’s IMPM with other areas of China.


AREA	SANMING	OTHER AREAS OF CHINA

Policy goals	Save medical insurance funds;Promote cooperation;Promote residents’ health	Save medical insurance funds;Promote cooperation;Promote residents’ health

The payment policies for ICHC		

(1) How to set GB?	According to population	According to population and ICHC’s medical insurance expenditure in previous years

(2) How to use GB?	ICHC owns the over-spending	ICHC shares the over-spending with medical insurance management institutions

	The balance can be used for institutional development, payment of doctor’ salary, cooperation among medical institutions, and health promotion	The balance can be used only for institutional development

The payment policies for medical personnel	Paid by ASS. The evaluation indexes of ASS were adjusted according to IMPM goals	Paid by volume of service. The volumes of service were adjusted according to IMPM goals

	Promoting the cooperation of medical institutions and residents’ health is one of the main evaluation criteria for medical personnel’s salary level	Even if they do not promote cooperation between medical institutions and residents’ health, medical personnel can get a high level of income


*Note:* This table illustrates the general situation only. Integrated payment policies in other parts of China may be quite different.

Firstly, the GB of ICHC in Sanming is determined according to population, while the GB of ICHC in other regions is determined according to population and ICHC’s medical insurance expenditure in previous years. Sanming stipulated that medical insurance fund balance can be used for institutional development, payment of doctor’s salary, cooperation among medical institutions, and health promotion (other regions only institutional development). Secondly, Sanming adjusts ASS assessment indicators of medical personnel according to IMPM objectives. Promoting the cooperation of medical institutions and residents’ health is one of the main evaluation criteria for medical personnel’s salary level. In other regions, medical personnel are paid according to the number of medical services. Even if they do not promote cooperation between medical institutions and residents’ health, medical personnel can get a high level of income.

## Description of the policy evaluation

### Selection of research cases

**Figure 1:**
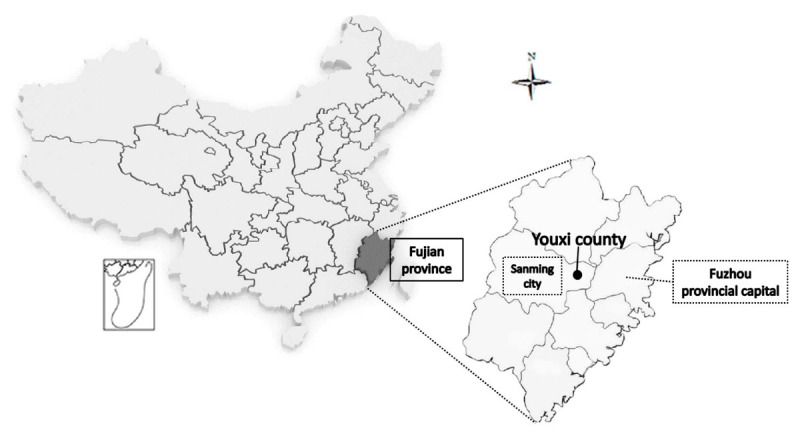
The geolocation of Youxi in Fujian Province, China.

There are 12 county-level cities in Sanming. Each county-level city must implement ICHC and IMPM simultaneously. The construction forms and integrated payment policies of 12 ICHCs are nearly the same. We select Youxi county of Sanming as the study case. (See Figure 1 and Table 2 for details of Youxi) Although from the perspective of the representativeness of the sample, there are limitations if only selects one sample. However, this study believes that using Youxi as a sample is more meaningful. Youxi implemented the IMPM policy in October 2017. At the beginning of the reform, the Sanming Municipal Government believed that if the reform has worked well in Youxi, where the policy implementation conditions are the worst, it will be more indicative that the reform policy is scalable. The success of IMPM in Youxi is also the reason why Sanming has comprehensively promoted the policy in the whole Sanming. The poor conditions in Youxi are reflected in two aspects: (1) In terms of traffic distance, Youxi is the closest county to Fuzhou city (the capital of Fujian Province) in Sanming. (See Figure 1 for details) (2) Before the reform, because it was the closest to Fuzhou, more residents in Youxi chose to go to the provincial capital hospital for treatment across the county. So the medical insurance expenditures of residents in Youxi took up the medical insurance funds of other counties every year, and there was a serious waste of resources.

**Table 2:** Social economic status and other characteristics of Youxi from 2016 to 2019.


INDICATORS	2016	2017	2018	2019

Total area (km^2^)	3463			

GDP (billion RMB)	20.1	23.1	24.6	26.3

Resident population (10000)	35.8	36.1	36.2	36.3

The proportion of the population aged 60 and above (%)	14.9	15.3	15.9	16.6

Per capita disposable income (RMB)	19687	21509	23067	25410

Number of CHs	3	3	3	3

Number of THs	18	18	23	23

Number of HCs	350	354	387	363

Number of ICHC	0	1	1	1

Number of health personnel per 1000	5.96	6.21	5.28	4.97

Number of practicing physicians per 1000	1.54	1.73	2.25	2.34

Number of registered nurses per 1000	1.83	2.01	2.32	2.63

Number of hospital beds per 1000	4.16	4.42	4.53	4.80


*Note:* GDP, Gross Domestic Product; RMB, Ren Min Bi.

### Data source and outcome variables

The data came from the database of Youxi’s Health Information System and special investigation. This study determines the outcome variables based on the implementation purpose of IMPM. It mainly includes: (1) Number of outpatient and emergency patients and hospitalizations of CHs; CHs outpatient and emergency expenses and hospitalization expenses; (2) Hospitalization rate of residents; (3) Number of healthcare outpatients in different hospitals and their proportion in the total; (4) Health management data, including standardized management rate and control rate of hypertension and diabetes.

The core purpose of the IMPM is to facilitate cooperation between different types of hospitals and to provide appropriate services as required by the government. In China, the function of CHs is to provide difficult medical services. The functions of primary healthcare institutions (THs/HCs) are to provide services such as common diseases, chronic diseases, and health promotion. Therefore, (1) firstly, we used CHs’ number of outpatient and emergency patients and number of hospitalizations to evaluate health care delivery, which can reflect whether the hospital has provided excessive medical services [20,21]. We mainly use the cost of outpatient and emergency patients per time to analyze the disease severity of CHs. The more serious the illness the hospital treats, the higher the cost is likely to be. (2) Secondly, we evaluated the changes in hospitalization rate of residents and the number of healthcare outpatient visits for different hospitals and their share of the total, which can reflect the provision of preventive services (such as health examinations, and health guidance). Such indicators can reflect whether different hospitals provide appropriate healthcare, and can also reflect the degree of cooperation between hospitals and primary healthcare institutions.

Promoting hospitals to place greater emphasis on health promotion programs is the goal of integrated payment incentives [22,23,24], which is the core purpose of the IMPM. Therefore, we evaluated health management indicators, which can reflect the effect of the measures taken by the ICHC of Youxi to promote health and reduce unnecessary medical services.

### Statistical analysis

This study used interrupted time-series analysis (ITSA) to assess the causal correlation of IMPM policies. However, because Youxi only collected annual data for some data, we could only conduct a chi-square test for appropriate annual data. (For example, the standardized management rate of hypertension in China is based on annual statistics, and monthly data cannot be collected.) Although the chi-square test results cannot be used as evidence of causal correlation, they can illustrate the correlation between annual data and time. During the study period (2016 to 2019), there was no major reform of the health system in Youxi and IMPM for ICHC was the most significant reform. Therefore, the results of annual data are used as auxiliary analysis results in this study, which aims to maximize the comprehensiveness of this study’s assessment of the effect of IMPM policy.

ITSA is a suitable evaluation approach when a single unit is being studied (that is, individual, city, state, country), when the outcome variable is serially ordered as a time series, and when multiple observations are captured in both the preintervention and postintervention periods [25,26]. ITSA has strong internal validity, even in the absence of a comparison group, primarily because of its control over the effects of regression to the mean [27]. Additionally, ITSA has strong external validity when the unit of measure is at the population level, or when the results can be generalized to other units, treatments, or settings [27]. After Gillings [28] used ITSA to study health services, ITSA has been widely used to assess the effectiveness of health policies [29]. ITSA uses baseline trends and levels to project future monthly outcomes with the assumption that these values reflect what would have happened without the policy (i.e., the counterfactual) [30]. The basic model includes terms that estimate the baseline level for each outcome (intercept), baseline trend (slope), change in the level of the outcome measured immediately after policy implementation, and change in post-policy trend [30].

When only one group is under study (i.e., no comparison groups), the regression model is expressed as,


{Y_t} = {\beta _0} + {\beta _1}(T) + {\beta _2}({X_t}) + {\beta _3}(X{T_t}),

Where *Y_t_* is the outcome variable during a period, which changes every month between February 2016 and November 2019, T is the time since the start of the study (February 2016 = 1, …, November 2019 = 46), and *X_t_* is a dummy (indicator) variable that represents the intervention. Pre-intervention periods are denoted as 0; otherwise, the value is 1. In this study, the value of *X_t_* before October 2017 is 0, whereas that after this period is 1. *XT_t_* is an interaction term, which is 0 before October 2017, and then increases by 1 each month from October 2017 (1 = October 2017, 2 = November 2017, 3 = December 2017, …). β_0_ represents the intercept or starting level of the outcome variable before the IMPM, *β*_1_ is the slope or trajectory of the outcome variable until the introduction of the IMPM, *β*_2_ is the level change following the intervention, and *β*_3_ indicates the slope change following the intervention. *XT_t_* is the interaction between time and intervention). The confidence interval of the P-value was 95%. Seasonal changes in the analysis data may skew the results. We handled the existence of seasonality through the moving average ratio method used in similar research [31]. Autocorrelation was assessed by examining the plot of residuals and the partial autocorrelation function, where data are normally distributed, conducting tests such as the BG test [32,33]. BG test suggested the existence of autocorrelations, which we corrected using the Regression with Newey-West standard errors [34].

### Study Results

#### Effect of medical service delivery for CHs in ICHC

Before the reform, CHs’ number of outpatient and emergency patients had an obvious upward trend (β1 = 578.3, P < 0.001); after the reform, the increasing trend of CHs’ number of outpatient and emergency patients slowed down significantly (β3 = –459.9, P < 0.001). Before the reform, CHs’ number of hospitalizations increased at a rate of 17.9 per month (β1 = 17.9, P < 0.001); after the reform, CHs’ number of hospitalizations decreased at a rate of 0.2 per month (β3 = –18.1, P < 0.001).

**Figure 2:**
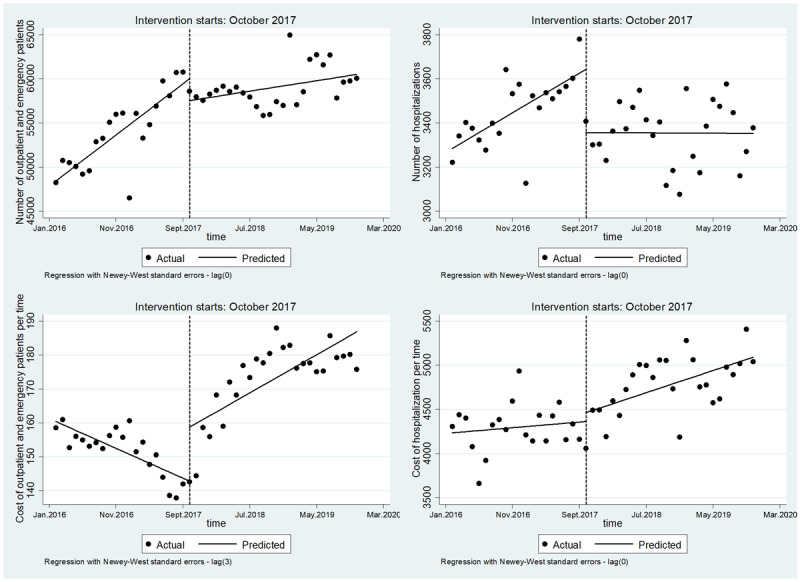
Outcome variables in CHs of ICHC.

Before the reform, the cost of outpatient and emergency patients per time of CHs decreased at a rate of 0.9 RMB per month (β1 = –0.9, P < 0.001); after the reform, the cost of outpatient and emergency patients per time of CHs increased at a rate of 1.1 RMB per month (β3 = 2.0, P < 0.001) (See Figure 2 and Table 3 for details).

**Table 3:** Interrupted time series analysis of outcome variables in CHS of ICHC.


OUTCOME VARIABLES	β_1, LEVEL CHANGE		β_2, LEVEL CHANGE		β_3, TREND/SLOPE CHANGE	
		
	P VALUE	(95% CI)	P VALUE	(95% CI)	P VALUE	(95% CI)

Number of outpatient and emergency patients	0.000	578.3 (462.5 to 694.1)	0.022	–2470.3 (–4572.9 to –367.7)	0.000	–459.9 (–596.5 to –323.3)

Number of hospitalizations	0.000	17.9 (11.7 to 24.2)	0.000	–286.0 (–406.8 to –165.1)	0.000	–18.1 (–26.8 to –9.4)

Cost of outpatient and emergency patients per time	0.000	–0.9 (–1.3 to –0.4)	0.008	15.9 (4.3 to 27.5)	0.000	2.0 (1.1 to 2.9)

Cost of hospitalization per time	0.451	6.4 (–10.6 to 23.4)	0.456	100.1 (–168.1 to 368.7)	0.083	18.7 (–2.5 to 39.9)


#### Effect of healthcare service delivery in Youxi

Table 4 shows the hospitalization rates and the number of healthcare outpatient visits in Youxi. From 2016 to 2019, the hospitalization rate in Youxi decreased from 17.0% to 16.3%. After Youxi implemented IMPM in October 2017, the number of healthcare outpatient visits in primary healthcare institutions increased from 682,000 in 2016 to 1.762 million in 2019, accounting for 68.5% of the total number of healthcare outpatient visits in Youxi, up from 50.8%. The index’s data difference was statistically significant (χ^2^_trend_ = 13.1, *P* < 0.05).

**Table 4:** Hospitalization rate and number of healthcare outpatient visits in Youxi.


VARIABLES	2016	2017	2018	2019

Hospitalization rate (%)	17	16.8	15.8	16.3

Number of healthcare outpatient visits (10k)	134.2	202.3	190.6	254.6

Proportion of hospitals (%)	49.2	37	39.2	30.8

Proportion of primary healthcare (%)	50.8	63	60.8	69.2


#### Effect of health management

Table 5 shows Youxi’s health management services data. After the reform of IMPM, the data of the four indicators showed an upward trend from 2017 to 2019 and had statistical significance. The hypertension standardized management rate increased from 69.9% to 81.5% (χ^2^_trend_ = 1063.1, *P* < 0.001). The hypertension control rate increased from 65.8% to 68.4% (χ^2^_trend_ = 222.6, *P* < 0.001). The diabetes standardized management rate increased from 71.3% to 78.3% (χ^2^_trend_ = 112.0, *P* < 0.001). The diabetes control rate increased from 65.0% to 72.5% (χ^2^_trend_ = 123.3, *P* < 0.001).

**Table 5:** Youxi’s health management data.


VARIABLES		2017	2018	2019

Hypertension	Standardized management rate (%)	69.9	78.4	81.5

	Control rate (%)	65.8	71.8	68.4

Diabetes	Standardized management rate (%)	71.3	76.9	78.3

	Control rate (%)	65.0	65.9	72.5


## Discussion

### CHs may have over-provided dispensable healthcare before IMPM reform

According to the ITSA results, the number of outpatient and emergency patients and hospitalizations at CHs showed an increasing trend before IMPM reform in October 2017. The reason may be that CHs have over-provided dispensable healthcare before IMPM reform. Although CHs in China are mandated by the government to treat serious diseases, primary healthcare institutions are mandated by the government to provide healthcare services. But before IMPM reform, Youxi’s hospitals were paid by FFS. Under FFS, however, a hospital’s income is proportional to the number of services it provides. The CHs in China have resources advantages such as high-level physicians, diagnosis and treatment technology, and large equipment, so it is easier to attract patients. To increase hospital revenue, CHs often attracted those patients who should be treated in primary healthcare to over-provided dispensable healthcare, which is a common problem in China [35]. Table 4 shows that in 2016, the hospitalization rate of Youxi reached 17.0% and CHs’ number of healthcare outpatient visits accounted for 49.2%, which may indicate the possibility of this phenomenon to some extent. At the same time, CHs’ cost of outpatient and emergency patients per time was declining before the IMPM reform, also due to CHs admitting too many patients who should be treated in primary healthcare. Because patients treated in primary healthcare usually have mild conditions and do not require many medical resources, the cost of their treatment is less expensive.

### CHs may reduce over-providing dispensable healthcare and cooperation between hospitals may increase after IMPM reform

Table 4 shows that the number of healthcare outpatient visits in Youxi increased from 1.342 million in 2016 to 2.546 million in 2019, indicating that the healthcare demand of residents in Youxi has a growing trend. However, according to the ITSA results, IMPM reform significantly slowed down the increasing trend of the number of outpatient and emergency patients and hospitalizations at CHs, and the monthly trends of these two indicators remained stable and fluctuated after October 2017. There may be three behaviors that cause this result, which are also the expected results of China’s medical reform and the reason why the Chinese government promotes the IMPM.

1. CHs tried not to attract patients who should be treated in primary healthcare after IMPM reform. Table 4 shows that from 2016 to 2019, the hospitalization rate decreased from 17.0% to 16.3% and the proportion of number of healthcare outpatient visits in CHs decreased from 49.2% to 30.8%, which may also reveal this phenomenon to some extent. At the same time, CHs’ cost of outpatient and emergency patients per time showed an upward trend after the IMPM. This change in trend may be because since CHs tried not to attract patients who should be treated in primary healthcare, which meant that CHs only retained severe patients. Severe patients usually consume higher medical resources and their costs of treatment are higher.
2. Collaboration between CHs and primary healthcare has increased. Before the IMPM reform, Chinese patients were used to going to CHs first for any illness, while the medical needs of Youxi residents were increasing. If CHs had not established a partnership with primary healthcare and had not referred patients with mild conditions to primary healthcare, it would have been difficult for CHs to maintain its monthly trend of number of outpatient and emergency patients and hospitalizations beyond IMPM.
3. The ICHC may pay more attention to the residents’ health. Health promotion is one of the goals of IMPM, and the Sanming Policy also provides clear guidance on this. Table 5 shows that the health management rate and control rate of hypertension and diabetes in Youxi increased from 2017 to 2019, which may be partly caused by CHS’ cooperation with primary healthcare to increase the support for chronic disease management.

## Lessen learned from Sanming’s IMPM

1. The GB is determined based on factors such as economic development in Sanming, and these factors are usually growing. ICHC in Sanming need not worry about the GB reduction. The policy mechanism of owning the over-spending means that ICHC will bear the consequences of overspending. At the same time, Sanming stipulates that a certain percentage of the medical insurance fund balance can be withdrawn as the salary of medical staff, which changes the salary incentive model of medical staff from “the more patients admitted and the more services they provide, the higher the salary” to “the lower the medical cost, the higher the salary.” These policies may increase the incentive for CHs to boost balances of medical insurance funds by cooperating with primary healthcare and increasing health promotion actions.
2. Different from other areas in China that only implement GB and no specific policy enforcement guidance, Sanming has innovatively formulated specific policy guidance on the use of medical insurance balance for cooperation between CHS and primary healthcare and health promotion projects. International studies have also shown the mechanisms of remaining the balance can help facilitate consolidation [36]. According to the theory of altruism and the rational economic man hypothesis [37,38,39], under specific policy enforcement guidance, managers and doctors usually tend to provide appropriate services, healthcare services rather than excessive medical services. Sanming’s specific policy guidance may serve two functions: prompt ICHC managers to adopt clear rules to facilitate collaboration between CHs and primary healthcare; allow ICHC managers to place greater emphasis on public health and preventive services than before and to take clear steps to promote healthcare integration, reduce disease morbidity, and save health care costs [15].
3. The research shows that the construction of medical staff’s compensation system and performance accountability system is the key factor to realize the integration of medical services [10,11,12]. Without the corresponding reform of the salary system, the regional medical insurance fund may still overspend [15]. According to the purpose of IMPM, Sanming adjusted ASS assessment indicators, adding cooperation indicators between CHs and primary healthcare (the proportion of number of healthcare outpatient visits in primary healthcare), and health promotion indicators (hypertension and diabetes control rate). These policies mean that the reform effect of IMPM directly affects the salary level of medical staff. Therefore, these policies may further strengthen the effect of IMPM.

However, in some parts of China, the GB is determined based on the medical insurance expenditure of ICHC in the previous year, which means that the less medical insurance expenditure in the previous year, the less GB in the current year. At the same time, the balance may be recovered by the medical insurance administration. ICHCs in these places usually maintain medical insurance expenditure at an overspending state to prevent medical income from falling and ensure that the GB does not fall in the current year. These policies will reduce CHs’ incentives to boost balances of medical insurance funds by cooperating with primary healthcare and increasing health promotion actions. At the same time, the government has not given similar policy guidance to Sanming, and the salary system of medical staff is still calculated according to the number of services. Even if medical staff do not promote the cooperation of medical institutions and residents’ health, medical personnel can get a high level of income. These policies may lead to CHs siphoning patients of THs (HCs) through the ICHC, and over-providing medical services to earn more medical funds [40].

## Limitations

(1) The IMPM studied in this paper is an integrated reform. Therefore, it is difficult to find areas with only a single reform as a control group to reflect the reform effect. (2) Due to the significant impact of COVID-19 on China in 2020 and 2021, the study period is determined to be 2016–2019. As part of the outcome variables in the study area are statistical in the form of annual data, this study can only conduct chi-square analysis and descriptive analysis for annual data, which cannot represent causal correlation. Follow-up studies should collect and analyze data over a long period time. (3) In China, outpatient and emergency care are counted together. Therefore, the data in this paper cannot distinguish between outpatient and emergency patients. (4) Sanming’s IMPM was implemented based on ICHC, while the effectiveness of IMPM implementation in other medical and health service systems remains uncertain.

## Conclusion

As a model promoted by the Chinese government, Sanming’s IMPM organically and systematically integrates multiple payment policies. Compared with other parts of China, the specific policies of Sanming’s IMPM are better matched with policy goals, which may be more conducive to promoting medical and health service providers to pay more attention to cooperation among medical institutions and population health.

## Abbreviations

FFS: Fee-For-Service

ICHC: Integrated County Healthcare Coalition

CHs: county-level hospitals

THs: township hospitals

HCs: health clinics

IMPM: Integrated Medicare Payment Methods

GB: global budget

ASS: annual salary system

ITSA: interrupted time series analysis
